# The Anorexigenic Peptide Neuromedin U (NMU) Attenuates Amphetamine-Induced Locomotor Stimulation, Accumbal Dopamine Release and Expression of Conditioned Place Preference in Mice

**DOI:** 10.1371/journal.pone.0154477

**Published:** 2016-05-03

**Authors:** Daniel Vallöf, Jesper Vestlund, Jörgen A. Engel, Elisabet Jerlhag

**Affiliations:** Institute of Neuroscience and Physiology, Department of Pharmacology, The Sahlgrenska Academy at the University of Gothenburg, Gothenburg, Sweden; University of Leicester, UNITED KINGDOM

## Abstract

Amphetamine dependence, besides its substantial economical consequence, is a serious cause of mortality and morbidity. By investigations of the neurochemical correlates through which addictive drugs, such as amphetamine, activate the mesoaccumbal dopamine system unique targets for treatment of drug addiction can be identified. This reward link consists of a dopamine projection from the ventral tegmental area to the nucleus accumbens (NAc) suggesting that these brain areas are important for reward. The physiological function of gut-brain peptides has expanded beyond food intake modulation and involves regulation of drug reinforcement. A novel candidate for reward regulation is the anorexigenic peptide neuromedin U (NMU). We therefore investigated the effects of intracerebroventricular (icv) administration of NMU on amphetamine’s well-documented effects on the mesoaccumbal dopamine system, *i*.*e*. locomotor stimulation and accumbal dopamine release in mice. In addition, the effect of accumbal NMU administration on locomotor activity was examined. The effect of NMU, icv or intra-NAc, on the expression of conditioned place preference (CPP) was elucidated. Firstly, we showed that icv administration of NMU attenuate the amphetamine-induced locomotor stimulation, accumbal dopamine release and expression of CPP in mice. Secondly, we found that a lower dose of NMU (icv) reduce the amphetamine-induced locomotor stimulation in mice. Thirdly, we demonstrated that NMU administration into the NAc block the ability of amphetamine to cause a locomotor stimulation in mice. However, accumbal NMU administration did not attenuate the amphetamine-induced expression of CPP in mice. Our novel data suggest that central NMU signalling is involved in development of amphetamine dependence.

## Introduction

Addiction to amphetamine, a common cause of mortality and morbidity, is a major cost for both the society and the individual [1, 2]. However, effective pharmacological interventions to treat amphetamine dependence are currently limited. Development of drug dependence, including amphetamine, is attributed to the ability of addictive drugs to interfere with the mesoaccumbal dopamine system consisting of midbrain dopaminergic neurons of the ventral tegmental area (VTA) projecting to the nucleus accumbens (NAc) (for review see [3, 4]). Through investigations of the neurochemical mechanisms involved in this activation, original treatment candidates for drug dependence can be developed. Over the last years a pivotal role of appetite regulatory peptides, such as ghrelin and glucagon-like peptide 1 (GLP-1), in the development of drug reinforcement has been identified (for review [5]). Given that appetite regulation is complex and involves numerous peptides the possibility should be considered that other gut-brain peptides, such as neuromedin U (NMU), could modulate reward.

The highly conserved neuropeptide NMU is detected along the gastrointestinal tract as well as in the brain. In the rat gut, NMU-like immunoreactivity has been demonstrated in nerve cell bodies as well as fibers in the submucosal and myenteric plexuses, as opposed to endocrine cells (for review see [6]). NMU acts via two distinct receptors namely NMUR1 and NMUR2 [7]. NMUR1 is expressed in a vast variety of peripheral tissues including the lungs and intestine (for review see [6]), as well as in the amygdala [8]. NMUR2 are predominantly expressed in the brain areas [8], albeit expression in peripheral tissues such as lungs and ovary has been identified [9]. The divers physiological role of peripheral NMU, via activation of predominantly NMUR1, involves nociception, smooth muscle stimulation, stress responses and body temperature regulation (for review [10]). However, in agreement with the high expression of NMUR2 along the gut-brain axis, research has shown that NMU serves as a catabolic signal via central mechanism. Indeed, central administration of NMU decreases whereas NMU antisense increases food intake in rats [11–15]. The anorexigenic properties of NMU are further strengthened by the findings that NMU overexpressing mice are hypophagic [16], whereas NMUR2 knockout mice display increased intake of high fat diet compared to wild type mice [17]. The anorexigenic properties of NMU are mediated via NMUR2 located in key energy balance regulating brain areas such as arcuate nucleus and paraventricular nucleus [13, 14, 18]. Data showing that NMU knockout mice display an increased body weight as well as adiposity [19] and that NMU overexpressing mice are lean [16] support a role for NMU in body weight regulation. In addition, sub-chronic central NMU administration reduces body weight and total energy intake in diet-induced obese mice [11, 12]. Despite the fact that NMUR2 are expressed in reward related areas such as NAc [8], that NMU immunoreactivity fibers are detected in the NAc and VTA [20, 21] and that NMUR2 knockdown rats display binge-type food consumption as well as an enhanced preference for higher-fat food [17], the role of NMU in drug induced reward has not been evaluated. We therefore investigate the possibility that NMU (icv) may serve as a regulator of amphetamine-induced activation of the mesoaccumbal dopamine system as measured by locomotor stimulation and accumbal dopamine release. Given the fact that amphetamine, at least in part, induces reward by reversing the reuptake of dopamine in NAc, an additional subject for the present study was to investigate the effect of intra-NAc infusion of NMU on the ability of amphetamine to cause a locomotor stimulation. In addition, we further investigated the effect of NMU, icv or intra-NAc, on the expression of conditioned place preference (CPP), a measure of reward-related contextual cues associated with drug experience and thus of importance for acquiring as well as maintaining drug taking behaviour.

## Materials and Methods

### Animals

Adult post-pubertal age-matched male NMRI mice (8–12 weeks old and 25–40 g body weight; Charles River, Susfeldt, Germany) were used. The mice were allowed to acclimatize at least one week before the start of the experiment and were group housed and maintained at a 12/12 hour light/dark cycle, a temperature at 20°C and a 50% humidity. Tap water and food (normal chow; Harlan Teklad, Norfolk, England) were supplied *ad libitum*. The Swedish Ethical Committee on Animal Research in Gothenburg approved the experiments and all efforts were made to minimize animal suffering as well as to reduce the number of animals used. Each experiment used an independent set of mice

### Drugs

For studies investigating amphetamine-induced activation of the mesoaccumbal dopamine system in mice, dex-amphetamine sulphate (RBI, Natick, USA) was dissolved in vehicle (0.9% sodium chloride solution) and was administered intraperitoneal (ip) at a dose of 2 mg/kg 10 minutes prior to initiation of the experiments. NMU (Bionuclear, Bromma, Sweden) was diluted in Ringer solution (NaCl 140 mM, Ca Cl_2_ 1.2 mM, KCl 3.0 mM and MgCl_2_ 1.0 mM; Merck KGaA, Darmstadt, Germany). A dose of 1 μg in 1 μl for intracerebroventricular (icv) administration was selected since this dose previously was found to reduce food intake in mice [12–14] and recently shown to attenuate alcohol mediated behaviours in rodents [22]. Our recent data showed that central administration of the selected dose of NMU (1 μg, icv) had no effect *per se* on locomotor activity, accumbal dopamine release and the expression of CPP in mice [22]. In addition, we used a lower dose of NMU (0.3 μg in 1 μl, icv) which has been shown to reduce alcohol intake in rats [22]. For local and bilateral administration into the NAc a dose repose study was conducted. A dose of 62.5 ng in 0.5 μl (per side) was selected since this dose had no effect *per se* on locomotor activity or the expression of CPP in mice.

### Guide cannula implantation

In order to administer NMU or vehicle solution guide cannulas (stainless steel, length 10 mm, with an o.d./i.d. of 0.6/0.45 mm) were implanted four days prior to the experiments. The surgery was conducted as follows: The rodent was anesthetized with isofluran (Isofluran Baxter; Univentor 400 Anaesthesia Unit, Univentor Ldt., Zejtun, Malta), placed in a stereotaxic apparatus (David Kopf Instruments; Tujunga, CA, USA) and kept on a heating pad to prevent hypothermia. Two drops of Xylocain adrenalin (5 μg/ml; Pfizer Inic; New York, USA) were used for local anaesthesia. The skull bone was exposed after an incision and three holes were drilled, two for the guide cannula and one for the anchoring screw. The coordinates for the third ventricle (for icv administration) relative to bregma were AP -0.9 mm and ML ±0.0. The coordinates for NAc were AP +1.4 mm and ML ±0.6. The guide cannulas were placed 1 mm below the surface of the brain and they were subsequently anchored to the screw and the skull bone with dental cement (DENTALON^®^ plus; Agntho’s AB, Lidingö, Sweden). After surgery the mice were injected with carprofen (Rimadyl^**®**^) (Astra Zeneca; Gothenburg, Sweden) at a dose of 5 mg/kg subcutaneously (sc) to relieve pain and were kept in individual cages (Macrolon III). At the time of the experiment, the cannula was extended another 1.1 mm or 3.6 mm ventrally beyond the tip of the guide cannula aiming for drug administration in the third ventricle and NAc respectively. One hour before initiating the experiment, a dummy cannula was carefully inserted and retreated into the guide cannula to remove clotted blood and hamper spreading depression. The drug was administered over one minute; the cannula was left in place for another minute and it was then retracted (5 μl Kloehn, microsyringe; Skandinaviska Genetec AB, V. Frölunda, Sweden). The injection sites were afterwards verified and only mice with correct placements were included in the statistical analysis.

### Locomotor activity experiments

Locomotor activity was performed as previously described [23]. The locomotor activity was registered in eight sound attenuated, ventilated and dim lit locomotor boxes (420 x 420 x 200 mm). In three of the tests, five by five rows of photocell beams (Kungsbacka mät- och reglerteknik AB, Fjärås, Sweden) allowed a computer-based system to register the activity of the mice by photocell detection. In these experiments locomotor activity was defined as the accumulated number of new photocell beams interrupted per 5 minutes. A second locomotor activity system (Open Field Activity System; Med Associates Inc, Gerogia, Vermont, USA) was used when investigating the effect of central (icv) administration of a low dose (0.3 μg) of NMU on amphetamine-induced locomotor stimulation. In this system 15 x 15 infrared beams allowed a computer-based system to register the distance travelled (cm per 5 minutes) by each mouse. In each experiment the mice were allowed to habituate to the locomotor activity box one hour prior to drug challenge. NMU was always administrated 20 minutes prior to amphetamine and the activity registration started ten minutes after the last injection. The locomotor activity was registered for an additional 60 minutes.

In the first experiment, the effects of NMU (1 μg, icv) on amphetamine-induced (2 mg/kg, ip) locomotor stimulation were investigated. Each mouse received one treatment combination (vehicle-vehicle, vehicle-amphetamine, NMU-vehicle or NMU-amphetamine) and was only subjected to one experimental trial.

In the second experiment, the effects of NMU (0.3 μg, icv) on amphetamine-induced (2 mg/kg, ip) locomotor stimulation were explored. Each mouse received one treatment combination (vehicle-vehicle, vehicle-amphetamine, NMU-vehicle or NMU-amphetamine) and was only subjected to one experimental trial.

The third experiment was conducted to establish a dose for intra-NAc administration without no effect per se on locomotor activity. The effects of local administration of vehicle or NMU (250, 125 or 62.5 ng in 0.5 μl per side) bilaterally into the NAc were studied in mice.

In the fourth experiment in separate mice the role of accumbal NMUR2 for amphetamine-induced reward was investigated. Therefore, the effects of intra-NAc NMU (62.5 ng in 0.5 μl) administration on amphetamine-induced (2 mg/kg, ip) locomotor stimulation were evaluated. Each mouse received one treatment combination (vehicle-vehicle, vehicle-amphetamine, NMU-vehicle or NMU-amphetamine) and was only subjected to one experimental trial.

### In vivo microdialysis and dopamine release measurements

The present experiment investigates the role of central NMU for amphetamine-induced activation of the mesoaccumbal dopamine system. For measurements of extracellular dopamine levels, mice were implanted with a microdialysis probe positioned in the shell of NAc. Surgeries were performed as previously described [23] and above (Guide cannula implantation). In these mice three holes were drilled, one for the probe, one for the guide cannula and one for the anchoring screw. The probes were custom made as described previously [24] and were randomly alternated to either the left or right side of the brain. The coordinates for the probes were 1.4 mm AP, ±0.6 ML and 4.7 DV mm [25].

The effect of central administration of NMU (1 μg, icv) on amphetamine-induced (2 mg/kg, ip) accumbal dopamine release was investigated using microdialysis in freely moving mice. On the day of the experiment the probe was connected to a microperfusion pump (U-864 Syringe Pump; AgnThós AB) and perfused with Ringer solution at a rate of 1.5 μl/minute. After one hour of habituation to the microdialysis set-up, perfusion samples were collected every 20 minutes. The baseline dopamine level was defined as the average of first three consecutive samples (-40 min until 0 min). After baseline samples, NMU or vehicle was administered (0 minutes). Amphetamine was administered at 20 minutes later (20 minutes), creating the following treatment groups: vehicle-amphetamine and NMU-amphetamine. Eight consecutive 20-minute samples were thereafter collected. The challenge-induced increase in accumbal dopamine was calculated as the percentage of increase from the baseline samples.

Dopamine was separated and quantified using two different high-performance liquid chromatography apparatuses with electro chemical detection as described previously [26]. In brief, a pump (UltiMate 3000 Pump; Thermo Scientific, Darmstadt, Germany), an ion exchange column (Nucleosil SA, 2.0 x 150 mm, 5 μm diameter, pore size 100 Å; Phenomenex Scandinavia, Västra Frölunda, Sweden) and a detector (Decade, Kovalent AB, Sweden) operated at 400 mV versus the cell were used. The mobile phase was delivered at 0.3 ml/min and consists of 58 mM citric acid, 135 mM NaOH, 0.107 mM Na2–EDTA and 20% methanol. The second system consisted of a pump (UltiMate 3000 Pump; Thermo Scientific, Darmstadt, Germany), a reversed phase column (2.0 x 50 mm, 3 μm diameter; pore size 100 Å; Phenomenex Scandinavia, Västra Frölunda, Sweden) and a detector (Dionex, Västra Frölunda, Sweden) operated at 220 mV versus the cell. The mobile phase was delivered at 0.3 ml/min and consists of f 150 mM NaH2PO4, 4.76 mM citric acid, 3 mM sodium dodecyl sulphate, 50 μM EDTA, as well as 10% MeOH and 15% acetonitrile.

### Conditioned place preference

The CPP experiments were designed to evaluate the effects of NMU, icv or accumbal, on the expression of amphetamine-induced CPP. The CPP test was performed in mice as previously described [23]. In brief, a 2-chambered conditioned place preference apparatus (45 lux) and distinct visual and tactile cues was used. The procedure consisted of preconditioning (day 1), conditioning (days 2 to 5), and postconditioning (day 6). At preconditioning mice were placed in the chamber with free access to both compartments during 20 minutes to determine the initial place preference. Conditioning (20 minutes per session) was done using a biased procedure in which amphetamine (2 mg/kg, ip) was paired with the least preferred compartment and vehicle with the preferred compartment. All mice received one amphetamine and one vehicle injection every day and the injections were altered between morning and afternoon in a balanced design. In the first experiment the mice were injected with NMU (1 μg, icv) or an equal volume of vehicle solution (Ringer) at postconditioning day. 20 minutes later the mice were placed on the midline between the two compartments with free access to both compartments for 20 minutes (creating the following treatment groups; amphetamine-vehicle and amphetamine-NMU). In the second experiment the mice were injected with NMU (62.5 ng) or an equal volume of vehicle solution (Ringer) bilaterally into the NAc at postconditioning day. 20 minutes later the mice were placed on the midline between the two compartments with free access to both compartments for 20 minutes (creating the following treatment groups; amphetamine-vehicle and amphetamine-NMU). CPP was calculated as the difference in percentage (%) of total time spent in the drug-paired (i.e., less preferred) compartment during the postconditioning and the preconditioning session. A control experiment for intra-NAc shell administration of NMU (62.5 ng in 0.5 μl per side) was also conducted. A separate mice group was subjected to the same procedure, but received vehicle injections instead of amphetamine throughout the conditioning (non-amphetamine conditioned control group; creating the following treatment groups; vehicle-vehicle and NMU-vehicle).

### Verification of probe and guide cannulas placement

**Fig 1:**
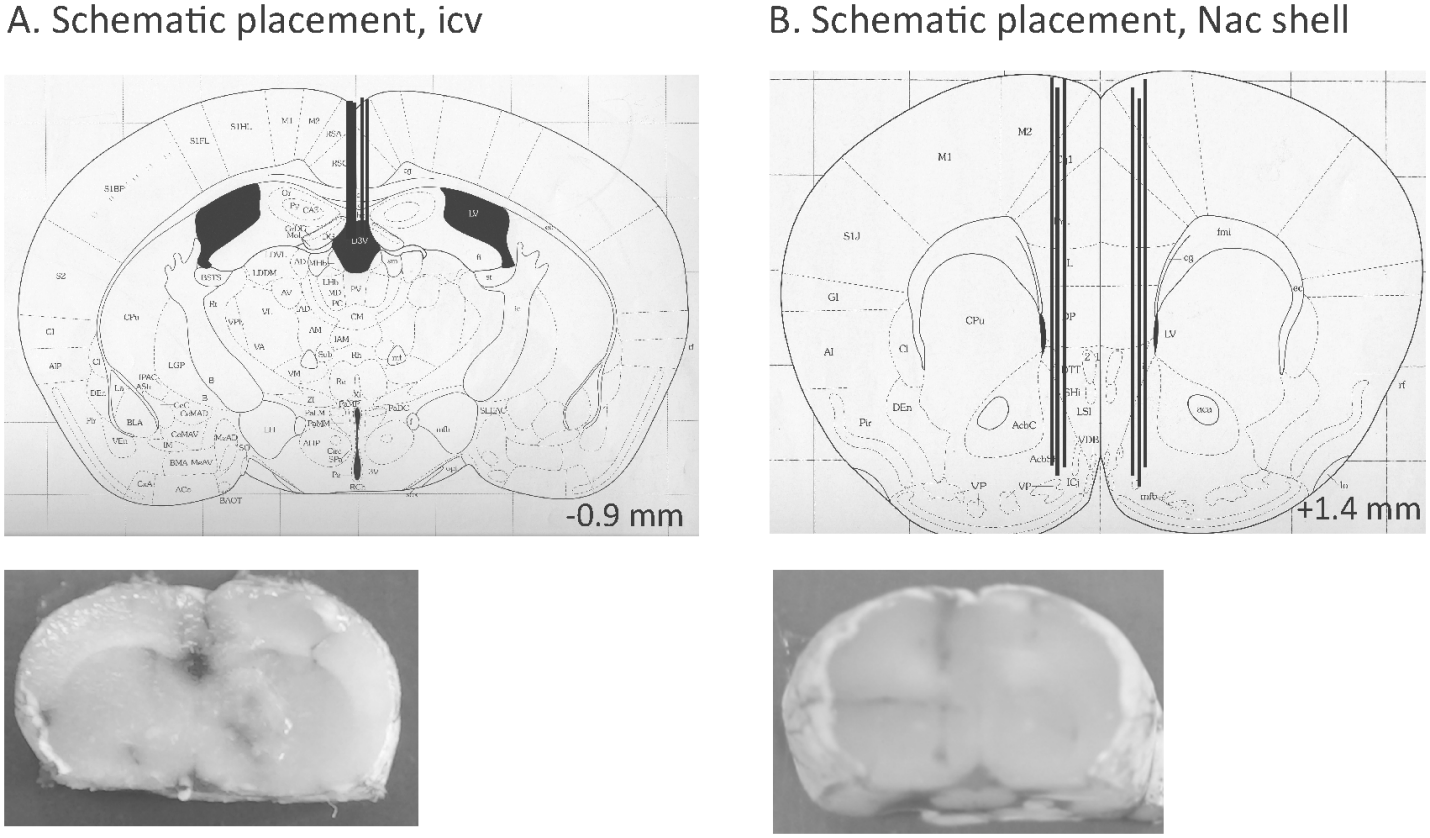
Schematic illustrations of placements. (A) A coronal mouse brain section showing five representative guide cannula placements (illustrated by vertical lines) aiming at the third ventricle (icv) [25]. In addition, a slice from mouse brain show one representative placement of a guide cannula in the third ventricle (B) A coronal mouse brain section showing six representative probe or guide cannula placements (illustrated by vertical lines) in the nucleus accumbens (NAc) shell [25]. Moreover, a slice from a mouse brain shows one representative placement in the NAc shell. For each brain section only a few representative placements are illustrated, but all other placements targeted the third ventricle or were within the NAc shell. Placements outside these areas were not included in the statistical analysis. The number given in the brain section indicates millimeters anterior (+) or posterior (-) from bregma.

Following each experiment the location of the probe (located in the NAc shell) and/or guide cannulas (located in the third ventricle (icv, Fig 1A) or in the NAc shell (Fig 1B)) was verified. The rodents were decapitated and the brains were mounted on a vibroslice device (752 M Vibroslice; Campden Instruments Ltd., Loughborough, UK). The brains were cut in 50 μm sections, and the location was determined [25] by observation using light microscopy. Only rodents with correct placement of the probe and/or guide cannulas were included in the statistical analysis. No animals were excluded due to severe illness. The only exclusion criterion was misplaced probes/guide cannulas.

### Statistical analysis

The locomotor activity experiments as well as microdialysis experiments were evaluated by a two-way ANOVA followed by Bonferroni post-hoc test for comparisons between different treatments and specifically at given time points. The CPP data were evaluated by an unpaired t-test. In addition, the effect of treatment on time spent in drug-paired compartment was analysed with a one-way ANOVA followed by a Bonferroni post-hoc test for comparisons between different treatment. Data are presented as mean ± SEM. A probability value of P<0.05 was considered as statistically significant.

## Results

### Effects of icv administration of NMU on amphetamine-induced locomotor stimulation, accumbal dopamine release and expression of CPP in mice

Analysis of baseline locomotor activity showed no overall main effect of treatment (F(_3,41_) = 1.18, *P* = 0.3306), time (F(_1,14_) = 0.68, *P* = 0.4236), or of treatment x time interaction (F(_3,42_) = 0.13, *P* = 0.9427), indicating that there are no differences in the baseline activity for the future treatment groups (vehicle-vehicle 508±42; vehicle-amphetamine 619±70; NMU-vehicle 563±52; NMU-amphetamine 691±40 counts per 60 minutes).

**Fig 2:**
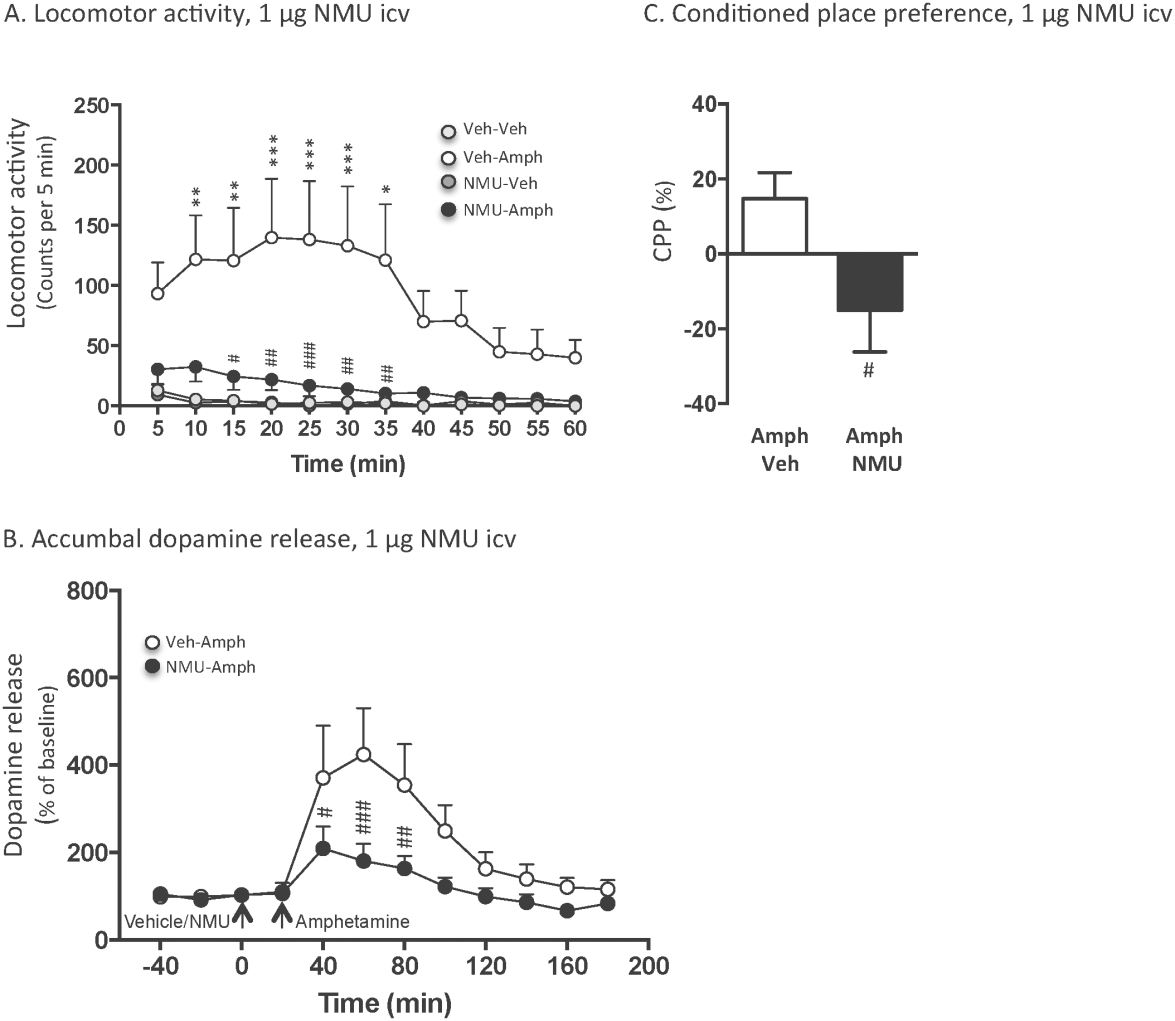
Central (1 μg, icv) administration of NMU attenuates amphetamine-induced locomotor stimulation, accumbal dopamine release and expression of conditioned place preference in mice. (A) Amphetamine-induced (2 mg/kg, ip) locomotor stimulation was blocked by a central injection of NMU (1 μg, icv) at time point 15–35 minutes. Central NMU administration had no effect *per se* on locomotor activity. (B) NMU (1 μg, icv) attenuated amphetamine (2 mg/kg, ip) induced increase in accumbal dopamine release at time point 40–80 minutes. (C) Central NMU (1 μg, icv, Amph-NMU) administration prevented the amphetamine (2 mg/kg, Amph-Veh) induced expression of conditioned place preference (CPP). Data are presented as mean ± SEM (*P<0.05, **P<0.01, ***P<0.001 for Veh-Veh versus Veh-Amph and # P<0.05, ## P<0.01, ### P<0.001 for Veh-Amph versus NMU-Amph).

An overall main effect of treatment (F(_3,252_) = 64.97, *P*<0.0001) as well as of time (F(_11,84_) = 2.02, *P* = 0.0358), but not of treatment x time interaction (F(_3,252_) = 0.97, *P* = 0.5209) was found on locomotor activity in mice following systemic administration of amphetamine (2 mg/kg) and central injection of NMU (1 μg, icv) (n = 8 for vehicle-vehicle, n = 8 for vehicle-amphetamine, n = 8 for NMU-vehicle as well as NMU-amphetamine). As shown in Fig 2A, post-hoc analysis revealed that amphetamine significantly increased locomotor activity compared to vehicle at 10–15 (*P*<0.01), 20–30 (*P*<0.001) as well as 35 (*P*<0.01) minute time points. This amphetamine-induced locomotor stimulation was significantly blocked by pre-treatment with icv injection of NMU at 15 (*P*<0.05), 20 (*P*<0.01), 25 (*P*<0.001) as well as 30–35 (*P*<0.01) minute time points. There was no difference in locomotor activity response in vehicle treated mice and NMU-amphetamine treated mice at any time point (*P*>0.05). The selected dose of NMU had no effect *per se* on locomotor activity compared to vehicle treatment at any time point (*P*>0.05).

Accumbal microdialysis measurements of dopamine in mice revealed an overall main effect of treatment (F(_1,117_) = 23.47, P<0.0001), time (F(_12,117_) = 5.415, P<0.0001) and of treatment x time interaction (F(_12,117_) = 2.209, P<0.0154) (Fig 2B). Post-hoc analysis revealed that icv NMU (NMU-amphetamine, n = 10) administration significantly attenuated the ability of amphetamine (vehicle-amphetamine, n = 10) to increase accumbal dopamine at time point 40 (P<0.05), 60 (P<0.001) and 80 minutes (P<0.01).

The amphetamine-induced (2 mg/kg) (amphetamine-vehicle, n = 8) CPP was significantly attenuated by an acute icv administration of NMU (1 μg, icv) (amphetamine-NMU, n = 7) on the post-conditioning day (*P* = 0.0361)(Fig 2C). One-way ANOVA analysis showed that there was an overall effect of treatment on time (sec) spent in the drug-paired chamber during pre- and post-conditioning (F(_3,26_) = 4.096, P = 0.0166). Bonferroni post-hoc test revealed that there was no difference in time spent in the least preferred compartment at preconditioning (vehicle 503±28 sec, NMU 523±28 sec, P>0.05). The time spent in the drug-paired compartment was higher in vehicle (680±60 sec) treated mice compared to NMU (343±122 sec) treated mice during post-conditioning (P<0.05).

Analysis of baseline locomotor activity showed no overall main effect of treatment (F(_3,42_) = 1.51, *P* = 0.2250), of time (F(_1,14_) = 2.52, *P* = 0.1347), or of treatment x time interaction (F(_3,42_) = 0.3985, *P* = 0.7548), indicating that there are no differences in the baseline activity for the future treatment groups (vehicle-vehicle 469±122; vehicle-amphetamine 590±141; NMU-vehicle 490±123; NMU-amphetamine 735±110 cm per 60 minutes).

**Fig 3:**
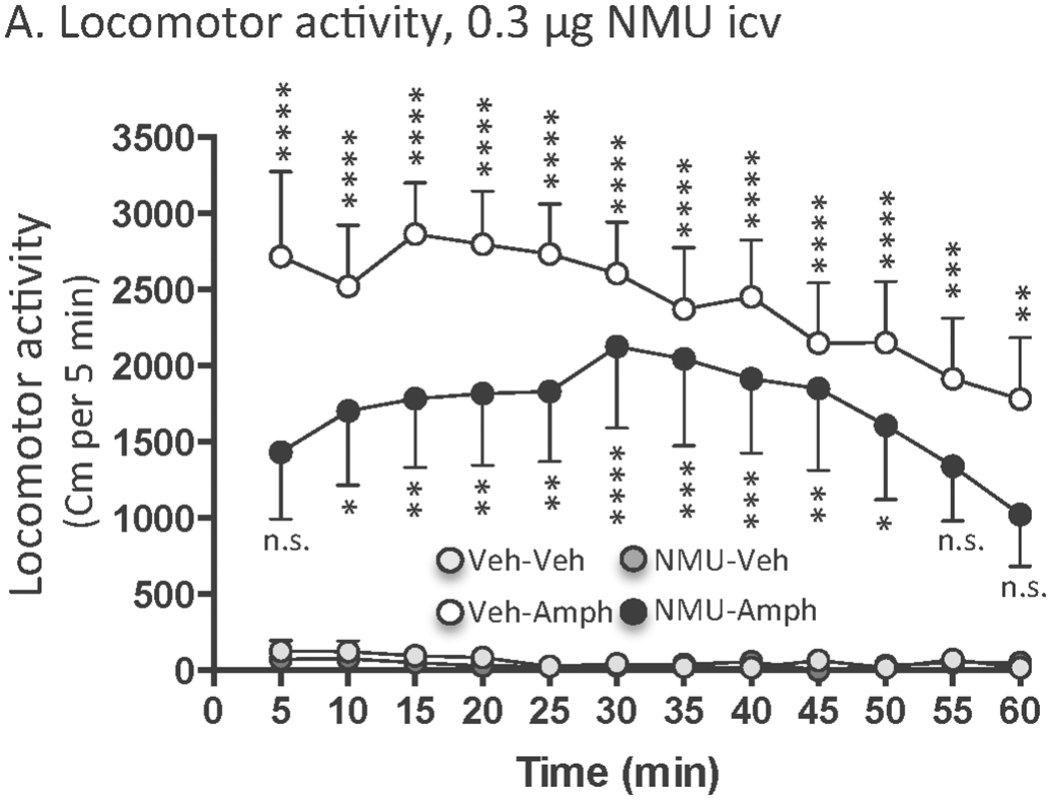
Central (0.3 μg, icv) administration of NMU reduces amphetamine-induced locomotor stimulation in mice. Amphetamine-induced (2 mg/kg, ip) locomotor stimulation was reduced, but not blocked, by a central injection of a lower does of NMU (0.3 μg, icv). Central administration of this lower dose of NMU had no effect *per se* on locomotor activity. Data are presented as mean ± SEM (n.s. P>0.05, *P<0.05, **P<0.01, ***P<0.001, ****P<0.0001 compared to vehicle-vehicle treatment).

An overall main effect of treatment (F(_3,252_) = 201.3, *P*<0.0001), but not of time (F(_11,84_) = 0.72, *P* = 0.7187) or of treatment x time interaction (F(_33,252_) = 0.53, *P* = 0.9856) was found on locomotor activity in mice following systemic administration of amphetamine (2 mg/kg) and icv injection of a low dose of NMU (0.3 μg) (n = 8 for vehicle-vehicle, n = 8 for vehicle-amphetamine, n = 8 for NMU-vehicle and n = 8 for NMU-amphetamine). As shown in Fig 3, post-hoc analysis revealed that amphetamine significantly increased locomotor activity compared to vehicle at 5–50 (*P*<0.0001), 55 (*P*<0.001) as well as 60 (*P*<0.01) minute time points. In addition, NMU-amphetamine treated mice showed an increase in locomotor activity compared to vehicle treated mice at 10 (*P*<0.05), 15–25 (*P*<0.01), 30 (*P*<0.0001), 35–40 (*P*<0.001), 45 (*P*<0.01) as well as 50 (*P*<0.05) minute time points. However, the NMU-amphetamine treated mice showed no significant response (*P*>0.05) to amphetamine at 5 as well as 50–60 minute time points. Indeed, the amphetamine response is lower in NMU-amphetamine treated mice compared to vehicle-amphetamine treated mice. NMU (0.3 μg, icv) had no effect *per se* on locomotor activity (*P*>0.05) compared to vehicle treatment at any time point. Collectively, these data show that a low dose of NMU reduces the amphetamine induced locomotor stimulation, but does not block the effect.

The locomotor activity data in Fig 2A are shown as counts per 5 minutes. The activity experiment shown in Fig 3 was analysed with a new system and therefore the data are presented as distance traveller per 5 minutes. Albeit two different systems were used, a robust amphetamine induced locomotor stimulation was obtained in both experiments.

### Effects of accumbal NMU administration on amphetamine-induced locomotor stimulation and on the expression of amphetamine-induced CPP in mice

Analysis of baseline locomotor activity showed no overall main effect of treatment (F(_3,42_) = 1.63, *P* = 0.1966), time (F(_1,14_) = 1.26, *P* = 0.2807), or of treatment x time interaction (F(_3,42_) = 0.11, *P* = 0.9913), showing that there were no differences in baseline activity in mice later on treated with various doses of NMU or vehicle locally into NAc (vehicle-vehicle 142±42; vehicle-amphetamine 130±33; NMU-vehicle 90±36; NMU-amphetamine 163±30 counts per 60 minutes).

There was an overall effect of treatment (F(_3,252_) = 15.18, *P*<0.0001), of time (F(_11,84_) = 5.55, *P*<0.0001) and of treatment x time interaction (F(_3,252_) = 2.45, *P*<0.0001) following bilateral administration of NMU (250, 125 or 62.5 ng in 0.5 μl per side) or vehicle into the NAc shell. Post-hoc test revealed that 250 ng NMU per side reduces the locomotor activity at the 5 (*P*<0.001), 10 and 20 (*P*<0.05) minute time points compared to vehicle. In addition, 125 (P<0.01) ng of NMU reduced locomotor activity compared to vehicle treatment 5 minute time point (*P*<0.001). NMU at a dose of 62.5 ng per side had no effect *per se* on locomotor activity in mice compared to vehicle treatment at any time point (*P*>0.05) (vehicle 191±46 n = 8; NMU250 43±20 n = 8; NMU125 23±17 n = 8; NMU62.5 137±23 n = 8 counts per 60 minutes).

Analysis of baseline locomotor activity showed no overall main effect of treatment (F(_3,66_) = 0.55, *P* = 0.6521), time (F(_1,22_) = 1.77, *P* = 0.1976), or of treatment x time interaction (F(_3,66_) = 0.08, *P* = 0.9714), indicating that there are no differences in the baseline activity for the future treatment groups (vehicle-vehicle 180±22; vehicle-amphetamine 130±20; NMU-vehicle 147±25; NMU-amphetamine 151±23 counts per 60 minutes).

**Fig 4:**
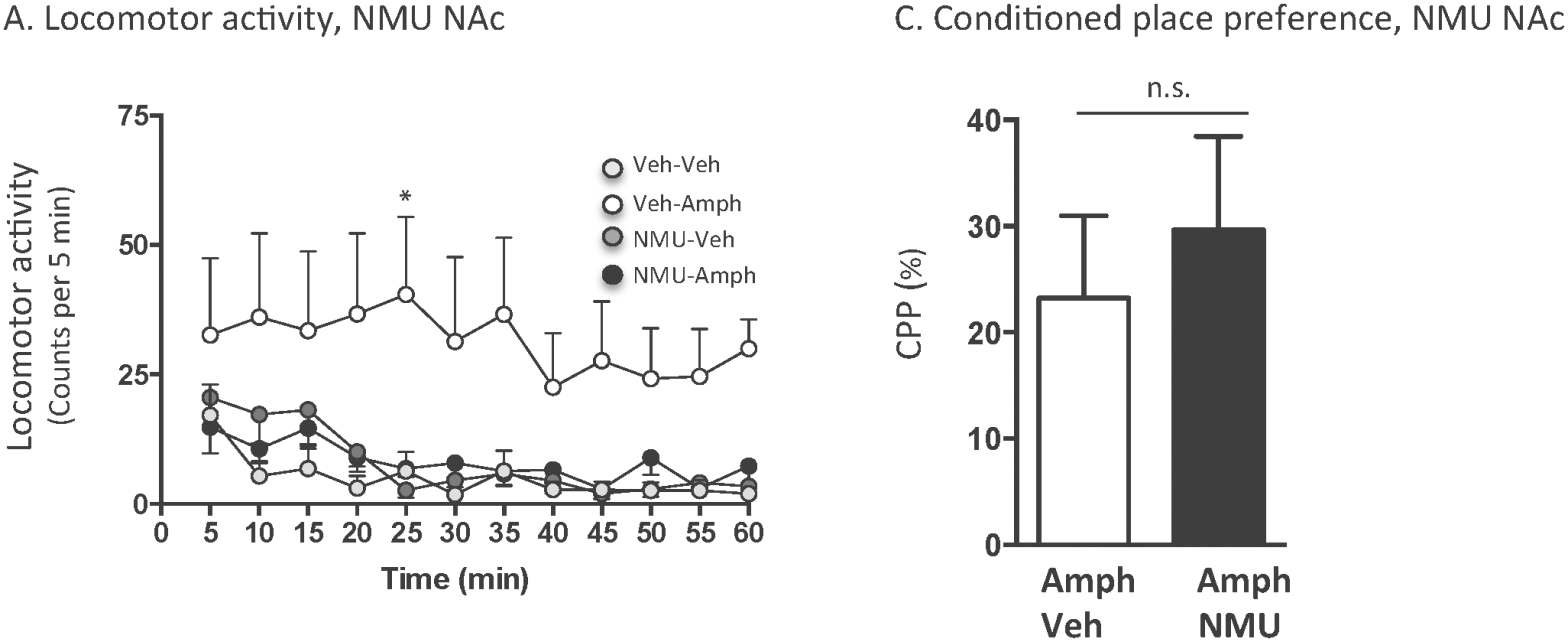
Accumbal administration of NMU attenuates amphetamine-induced locomotor stimulation, but does not affect the expression of conditioned place preference in mice. (A) Amphetamine-induced (2 mg/kg, ip) locomotor stimulation was attenuated by an accumbal injection of NMU (62.5 ng per side). Accumbal NMU administration had no effect *per se* on locomotor activity. (B) Accumbal NMU (62.5 ng per side, Amph-NMU) administration did not attenuate amphetamine-induced (2 mg/kg, ip, Amph-Veh) expression of CPP in mice. Data are presented as mean ± SEM (n.s. P>0.05, *P<0.05).

An overall main effect of treatment (F(_3,396_) = 36.70, *P*<0.0001), but not of time (F(_11,132_) = 1.14, *P* = 0.3387), or of treatment x time interaction (F(_33,396_) = 0.28, *P* = 0.9999) was found on locomotor activity in mice following systemic administration of amphetamine (2 mg/kg) and intra-NAc injection of NMU (62.5 ng /side) (n = 12 for vehicle-vehicle, n = 12 for vehicle-amphetamine, n = 12 for NMU-vehicle and n = 12 for NMU-amphetamine). As shown in Fig 4A, post-hoc analysis revealed that amphetamine significantly increased locomotor activity compared to vehicle at 25 (*P*<0.01) minute time point. There was no difference in locomotor activity response in vehicle treated mice and NMU-amphetamine treated mice at any time point (*P*>0.05). NMU into NAc had no effect *per se* on locomotor activity (*P*>0.05) compared to vehicle treatment at any time point.

Mice receiving bilateral vehicle administration into NAc (Fig 4A), compared to the third ventricle (Figs 2A and 3), showed lower locomotor activity as well as response to amphetamine. This could be explained by the fact that bilateral administration affects behaviour more than icv injections. However, this does not interfere with the interpretation of the data since vehicle controls are treated similarly to mice with drug challenges. A NMU dose response study was conducted in icv, but not intra-NAc, treated mice. The rational for this is that the amphetamine response is lower in intra-NAc vehicle treated mice and it would therefore be more difficult to differentiate a NMU recued amphetamine response, by a yet lower NMU dose, located between the vehicle-amphetamine and vehicle-vehicle treated mice (Fig 4A).

The amphetamine-induced (2 mg/kg) (amphetamine-vehicle, n = 11) expression of CPP was not affected by intra-NAc administration of NMU (62.5 ng/side) (amphetamine-NMU, n = 14) on the post-conditioning day (*P* = 0.5989)(Fig 4B). Separate experiment showed that bilateral administration of NMU into NAc shell did not induce a CPP *per* se (vehicle-NMU, 14 ± 15%; vehicle-vehicle, 5 ± 13%; *P* = 0.6639, n = 7 in each group).

## Discussion

The present study revealed three principal sets of findings. Firstly, we show for the first time that activation of the central NMU receptor signalling system inhibited the well-documented effects of amphetamine on the mesoaccumbal dopamine system [27], namely locomotor stimulation and accumbal dopamine release. In support for a modulatory role of NMU in reward processes are the previous data demonstrating that icv administration of NMU attenuated alcohol-induced locomotor stimulation, CPP and dopamine release as well as decreased alcohol intake in rodents [22]. In addition, NMU centrally reduced the intake of palatable food and increased consumption of standard chow [12] and paraventricular NMUR2 knockdown increased binge-eating behaviour as well as preference for palatable high fat diet [17]. The findings of a genome-wide allelic association study showed that polymorphisms in the NMUR2 gene are associated with alcohol use disorders [28] suggest that the presented data can be translationally valid. Furthermore, we found that administration of a lower dose of NMU into the third ventricle reduced, but did not block, the amphetamine induced locomotor stimulation in mice. In support for a dose dependent effect of NMU on reward regulation are the findings showing that central injection of this low NMU dose decreased alcohol intake in rodents [22]. When administrating a pharmacological agent into the third ventricle the possibility that the effects are due to intra-parenchymal mechanisms should be considered. Therefore, a limitation with the present study is that site-specific effects of icv-NMU could not be determined. We recently showed that the selected dose of NMU had no effect *per se* on locomotor activity (60 minutes following injection), accumbal dopamine release (100 minutes following injection) and the expression of CPP in mice [22], suggesting that the ability to block amphetamine reward is selective to activation of NMUR rather than other interfering systems. In consistency, supporting data show that central NMU administration does not affect the dopamine levels in brain areas including the NAc and does not affect forward locomotion (i.e. total transit time) in rats [8]. It should however be mentioned that higher doses of NMU increase locomotor activity in mice [29]. The possibility should also be considered that NMU could potentiate amphetamine-induced stereotypic behaviour and thereby decreasing total locomotion. Nevertheless, this appears less likely since our observational studies show that NMU does not alter gross behaviour in mice.

Secondly, we demonstrated that NMU administration into the NAc blocked the ability of amphetamine to induce a locomotor stimulation, suggesting that accumbal NMUR2 modulate amphetamine-induced activation of the mesolimbic dopamine system. Supportively preclinical findings show: i) amphetamine-induced locomotor stimulation is mediated, at least in part, via its ability to increase accumbal dopamine releases [30], ii) the behavioural effects of amphetamine are closely time-locked with accumbal dopamine release [31], iii) the mechanism of action of amphetamine includes reversal of the dopamine reuptake pump. Consistent with this concept are the data showing that the highest levels of NMU immunoreactivity are detected in the NAc [20]. Moreover, the detected distribution of immunoreactive accumbal NMU fibers [32] and the expression of NMUR2 has been identified in NAc in rats as well as in humans [8, 21]. Furthermore, central NMU administration activates *c-fos* expression in NAc [8]. The findings that intra-NAc NMU did not attenuate amphetamine-induced CPP raises the possibility that the obtained results may be influenced by tissue damages caused by local administrations. However, this appears less likely since we see that higher doses of accumbal NMU reduced the locomotor activity compared to vehicle treatment as well as that intra-NAc NMU significantly attenuated the amphetamine-induced locomotor stimulation. In addition, observations of the brain tissue show no obvious sign of tissue damage. In support for a local effect of NMU in the NAc are the previous data showing that administration of ghrelin into the NAc increases palatable food intake, but does not regulate sexual behaviour [33].

Thirdly, we showed in mice that icv administration of NMU, but not directly into the NAc, attenuates the expression of CPP that may reflect a measure of reward-related contextual cues associated with a drug experience [34]. In support are the recent data showing that icv NMU administration attenuated alcohol-induced expression of CPP in mice [22]. Collectively, the present data suggest that NMU, via central unknown mechanism, may alter the acquiring as well as maintaining of drug taking behaviour in mice. The expression of CPP induced by amphetamine depends upon a functioning mesocorticolimbic dopamine system, in particular the release of dopamine in NAc as well as in the prefrontal cortex (for review see [35]). In addition, the amphetamine induced CPP involves serotonin signalling in particular serotonin 2A/2B/2C receptors as well as the serotonin reuptake pump (for review see [35]). We here report that intra-NAc administration of NMU does not block the expression of CPP. One tentative explanation might be that NMUR2 in the prefrontal cortex or alteration of serotonergic signalling may be involved in the ability of amphetamine to elicit the expression of CPP. Consistently, icv NMU administration increases serotonin levels in brain areas such as the frontal cortex [8]. It should also be considered that NMUR2 in other brain areas, such as the arcuate nucleus and paraventricular nucleus, are important for amphetamine-induced activation of the mesoaccumbal dopamine system since both pharmacological and genetic studies collectively show that the anorexigenic properties of NMU involves NMUR2 in the arcuate nucleus and paraventricular nucleus [13, 14, 18]. Moreover, anti-NMU IgG reduces NMU-induced increased *c-fos* expression in paraventricular nucleus [36], suggesting that NMU targets the paraventricular nucleus directly. On the same note, arcuate nucleus and paraventricular nucleus, which both contain a high density of NMUR2, are highly interconnected [37] and they regulate the activity of mesolimbic structures via endorphinergic projections [38].

The downstream mechanisms through which central NMU signalling reduces amphetamine-induced reward remain unknown and need to be further investigated. The possibility that NMU attenuates amphetamine-induced reward is secondary to its effect on the hypothalamus-pituitary-adrenal (HPA) stress axis should be deliberated. Indeed, central administration of NMU, in higher doses than presently used, increases stress-like behaviours such as face washing, scratching and grooming [8] as well as elevates the plasma levels of the stress hormones ACTH and corticosterone [36]. Moreover, the NMU induced stress response is mediated via corticotrophin-releasing hormone [29]. Corticotrophin-releasing hormone knockout mice are unresponsive to NMU’s anorexigenic effects [39] and corticotrophin-releasing hormone has been attributed a key role for the etiology as well as maintenance of drug addiction [40]. We however hypothesize that NMU mediated reward does not involve stress repose since it has been shown that the selected dose, as opposed to higher doses, of icv NMU does not affect corticosterone levels in rodents [8, 22, 36]. The findings that local administration of NMU into the paraventricular nucleus causes a release of corticosterone as well as induces grooming, may indicate that icv administration of the selected dose of NMU does not reach deeper brain areas such as the paraventricular nucleus and therefore does not activate the HPA axis. In addition, the possibility that the selected dose of NMU induces an unmeasured stress response should also be considered. Findings show that central administration of NMU, dose-dependently, increases the oxytocin plasma levels [36] as well as c-Fos in oxytocin-immunoreactive neurons in the paraventricular nuceus [41] and that exogenous administration of oxytocin attenuates drug reinforcement (for review see [42]). These data provide oxytocin as a tentative downstream target for NMU regulated activation of the mesolimbic dopamine system.

During the last years the traditional role of gut-brain peptides as modulators of energy homeostasis has been extended and it has been shown that several of these peptides regulate reward (for review see [5]). Pharmacological or genetical suppression of the receptor for the orexigenic peptide ghrelin attenuates the ability of several addictive drugs to activate the mesoaccumbal dopamine system (for review see [5]). In support of this contention is that peripheral administration of glucagon-like peptide-1 receptor agonist, a satiety hormone, blocks reinforcement in rodents [43–47]. Moreover, gut-brain peptides such as orexin, galanin, cholecystokinin and leptin regulate drug reinforcement in rodents [48–51].

Collectively the present series of experiments show that central NMU signalling regulates amphetamine-induced reward as well as reward-related contextual cues associated with drug experience in mice. Moreover, accumbal NMUR2 regulate amphetamine induced locomotor stimulation but not the expression of CPP in mice. We therefore argue that the role of central NMUR2 in drug dependence should be investigated further. Nevertheless, the multiple functions of NMU should be taken into account when considering targeting NMU signalling for treatment of drug addiction.
